# Linear B-Cell Epitopes in Human Norovirus GII.4 Capsid Protein Elicit Blockade Antibodies

**DOI:** 10.3390/vaccines9010052

**Published:** 2021-01-14

**Authors:** Hassan Moeini, Suliman Qadir Afridi, Sainitin Donakonda, Percy A. Knolle, Ulrike Protzer, Dieter Hoffmann

**Affiliations:** 1 Institute of Virology, School of Medicine, Technical University of Munich, 81675 Munich, Germany; suliman.afridi86@gmail.com (S.Q.A.); protzer@tum.de (U.P.); dieter.hoffmann@tum.de (D.H.); 2 Institute of Molecular Immunology, School of Medicine, Technical University of Munich, 81675 Munich, Germany; sainitin.donakonda@tum.de (S.D.); percy.knolle@tum.de (P.A.K.)

**Keywords:** norovirus, GII.4 genotype, molecular dynamic simulation, linear blockade epitope

## Abstract

Human norovirus (HuNoV) is the leading cause of nonbacterial gastroenteritis worldwide with the GII.4 genotype accounting for over 80% of infections. The major capsid protein of GII.4 variants is evolving rapidly, resulting in new epidemic variants with altered antigenic potentials that must be considered for the development of an effective vaccine. In this study, we identify and characterize linear blockade B-cell epitopes in HuNoV GII.4. Five unique linear B-cell epitopes, namely P2A, P2B, P2C, P2D, and P2E, were predicted on the surface-exposed regions of the capsid protein. Evolving of the surface-exposed epitopes over time was found to correlate with the emergence of new GII.4 outbreak variants. Molecular dynamic simulation (MD) analysis and molecular docking revealed that amino acid substitutions in the putative epitopes P2B, P2C, and P2D could be associated with immune escape and the appearance of new GII.4 variants by affecting solvent accessibility and flexibility of the antigenic sites and histo-blood group antigens (HBAG) binding. Testing the synthetic peptides in wild-type mice, epitopes P2B (336–355), P2C (367–384), and P2D (390–400) were recognized as GII.4-specific linear blockade epitopes with the blocking rate of 68, 55 and 28%, respectively. Blocking rate was found to increase to 80% using the pooled serum of epitopes P2B and P2C. These data provide a strategy for expanding the broad blockade potential of vaccines for prevention of NoV infection.

## 1. Introduction

Noroviruses cause most of acute gastroenteritis cases in children and adults worldwide [1]. According to recently published reports, norovirus causes 685 million cases of cute gastroenteritis every year, resulting in a total cost of about $60 billion worldwide [2,3,4]. Norovirus is highly infectious and can be serious to individuals with underlying conditions, the elderly, and young children [1]. Noroviruses are genetically grouped into ten genogroups (GI–GX) with GI, GII, and GIV causing human infections [5,6,7]. While there is substantial diversity among noroviruses, genotype GII.4 accounts for over 80% of human NoV infections annually [8]. In GII.4 variants, the P2 domain, the most surface-exposed part of the capsid protein, evolves rapidly by frequent amino acid substitutions, resulting in the emergence of new epidemic variants as well as broad genetic and antigenic diversity of circulating norovirus variants [9,10,11,12]. Through high evolutionary rate, new antigenic variants emerge from the GII.4 lineage every 2–3 years and are often associated with widespread outbreaks [13]. This diversity poses potential challenges in development of a protective vaccine. On the other hand, the study of neutralization antibodies and epitopes is restricted by the lack of cell culture and animal models for HuNoV cultivation. To circumvent this problem, in vitro surrogate neutralization assays have been developed and bioinformatics tools were applied for prediction of blockade epitopes.

Previous studies described three conformational epitopes A, D, and E within the P2 domain of the GII.4 capsid protein [14,15]. In particular, residues 296–298 (epitope A) and 393–395 (epitope D), as well as surface residues at 333, 340, 356, 368, 372, 407, and 412–413 has been described as potential putative epitopes that change between epidemic GII.4 variants [9,10,11,14,16]. However, only a few studies have shown experiential evidence mapping GII.4 antigenic changes. For example, Allen et al. [14] identified conformational epitopes composed of residues 294–296 and 393–395 with comparing the reactivity of monoclonal antibodies with a 2002 epidemic GII.4 variant.

Peptide-based vaccines currently represent a novel strategy for addressing challenges associated with the prevention of viral infections. In this way, in silico analysis may apply to predict new B- and T-cell epitopes for in vivo applications. This approach has the ability to incorporate several epitope peptides directed against multiple virus strains into one single delivery system to induce broad immune responses in a single administration. In this study, we described linear antigenic epitopes which can elicit blocking antibodies against NoV GII.4 genetotype in vaccinated mice. Molecular dynamics (MD) simulation has been widely used in structural biology investigations and can serve as a powerful tool to determine the atomic-level behavior of biological events, such as protein conformational changes under different physiological conditions [17]. We applied structural modeling and MD simulation to realize how linear B-cell epitopes evolve over time and to gain a better understanding of the evolutionary dynamics of prevalent variants. The results help us understand the evolution of antigenic epitopes within the capsid protein, which is critical to the development of effective vaccines.

## 2. Materials and Methods

### 2.1. Capsid Protein Sequence Analysis

**Figure 1:**
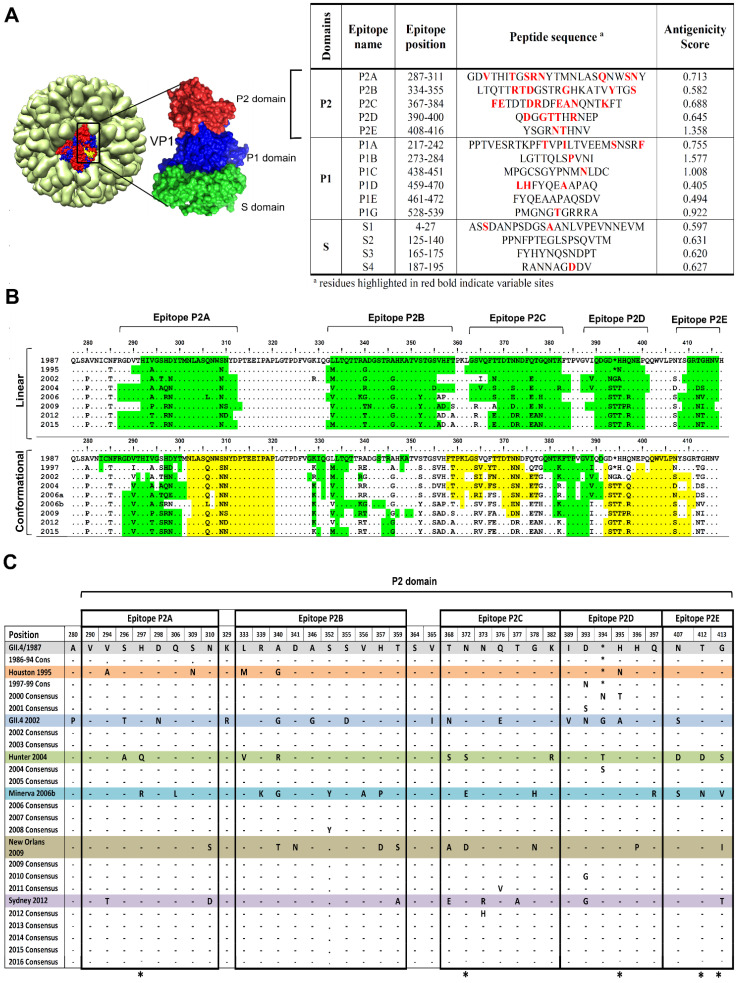
Predicted B-cell epitopes in NoV GII.4 variants and their evolutionary pattern. (**A**) Linear B-cell epitopes were predicted within norovirus Sydney GII.4 capsid protein using IEDB, Epitopia27 and BepiPred prediction tools: epitopes with antigenicity index higher that 0.4 were considered as antigen. NoV crystal structure (1IHM) was taken from protein data bank and structure was generated with chimera tool. (**B**) Evolutionary pattern of the predicted linear and conformation epitopes was determined within the GII4 variants connected with norovirus outbreaks. Yellow and green colors have been used to differentiate closed neighboring conformational epitopes (**C**) Columns of heterogenecity within P2 domain. Respective variants constituted yearly consensus sequences till they were replaced by the next variant. Positively selected sites are labelled with an asterisk below the columns.

A total of 600 HuNoV GII.4 capsid protein sequences available in GenBank were collected. For each year, a phylogenetic tree was first constructed with maximum likelihood algorithm in the MEGA7.0.14 package (Research Center for Genomics and Bioinformatics, Tokyo Meteropolitan University, Hachioji, Japan) [18]. Based on the phylogenetic tree and the sequence of the gene, sequences with very high similarity to other sequences grouped in the same cluster were removed and a consensus sequence was generated. The representative consensus sequences (listed in Figure 1C) along with the capsid sequence of the GII.4 variants associated with NoV outbreaks during the period 1987–2016 were aligned and amino acid variation within the P2 sub-domain was analyzed. Site-specific positive selection was assessed by computing nonsynonymous or synonymous substitutions in multiple GII.4 alignments capsid gene sequences. Our analysis was performed using the maximum likelihood ancestor counting method as available in the HyPhy package (Institute for Genomics, and Evolutionary Medicine, Temple University, PA, USA). The Tamure-Nei substitution model was chosen, and a neighbor-joining tree was used as an input.

### 2.2. Linear and Conformational B-Cell Epitope Prediction

Linear B-cell epitopes were predicted using the Immune Epitope Database Analysis Resource (IEDB), Epitopia27 (Artificial intelligence laboratory, Iowa state university, Ames, IA, USA) and BepiPred epitope prediction tools (Department of Bio- and health informatics, Technical University of Denmark, Copenhagen, Denmark) [19,20]. Potential spatial epitope positions (conformational epitopes) were predicted with the prediction tools available at the Conformational B-cell Epitope Prediction (CBTOPE) and Immune epitope database and analysis resource (IEDB) (tools.immuneepitope.org/ellipro) [21,22]. Antigenicity index of the predicted epitopes was calculated with VaxiJen v2.0 (The Jenner Institute, Oxford University, Oxford, UK) [23]. Antigenic indices were compared between the years using the antibody epitope prediction server IEDB Analysis Resource [24], based on the Kolaskar & Tongaonkar antigenicity scale [25]. From IEDB analysis resource, Emini Surface Accessibility Prediction and the Karplus & Schulz flexibility Prediction tools [26,27] were applied for computing the surface accessibility and residue flexibility of the capsid protein residues.

### 2.3. Structure Modelling and Epitope Mapping

Capsid protein secondary structures were predicted using highly accurate prediction tools Jpred 4 (Division of Computional Biology, University of Dundee, Dundee, UK) and PSIPRED (Protein Bioinformatic Group, University of Warwick, Coventry, UK) [28,29]. Homology 3-D models were generated for the reference sequences using I-TASSER 5.0 server (Yang Zang Research group, University of Michigan, Ann Arbor, MI, USA) [30]. Protein models were visualized in UCSC Chimera software (UCSF Resource for Biocomputing, Visualization and Informatics, San Francisco, CA., USA) [31]. The X-ray crystal structure of GII.4 major capsid protein VP1 (Protein Data Bank (PDB) accession no. 1Ihm) was used as a template. Structural alignment between the models and real capsid protein crystal structure was computed to measure the distance between the corresponding residues using Pymol (Schrödinger Inc., New York, NY, USA). The structural models were optimized using two energy minimization servers Modrefiner (PMID: 22098752) (Yang Zang Research group, University of Michigan, Ann Arbor, MI, USA) and Chiron tools (PMID:21058396) (Department of Biochemistry and Molecular Biology, Penn State Neuroscience Institute, Hershey, PA, USA); quality of the models was evaluated by PROSA (Center of Applied Molecular Engineering, Salzburg, Austria) [32] and Ramachandran plots were generated using Rampage server (http://mordred.bioc.cam.ac.uk/~rapper/rampage.php). Putative B-cell epitopes and variable sites that were potentially exposed on the surface of the capsid were mapped on the structures and analyzed for conformation changes over time from.

### 2.4. Molecular Dynamics Simulation Analysis

Molecular dynamics (MD) simulation was conducted using GROMACS 4.5 package (Science for Life laboratory, Stockholm and Uppsala, Sweden) [33]. The predicted 3-D structures were engaged in a cubic water box using the explicit transferable intermolecular potential with 3 points (TIP3P) water model at a buffering distance of 1.2 nm. The complexes were then subjected to minimization procedure with the Steepest Descent method for 2000 steps. All the simulations were run in periodic boundary conditions with the constant number pressure and temperature (NPT) ensemble. The Berendsen coupling algorithm was used for this process, and the temperature was kept at a constant 300 K with pressure at 1 bar. The Particle-Mesh Ewald (PME) method was applied to calculate electrostatic interactions with an interpolation of order 4 and grid spacing of 0.12 nm, and all bonds were constrained using the LINCS algorithm. Finally, the time step for all simulations was set at 2 fs, and 20 ns of MD simulation was performed. MD trajectories were analyzed with built-in GROMACS tools. Using the program gmx rms, the root mean standard deviation (RMSD) of the simulations was determined to assess the stability and equilibration of the VP1 P2 domain. Flexibility in an indicator for potential antigenic sites, as flexible regions can adjust their conformation upon interaction with antibodies [34]. To understand the contribution of each residue to the flexibility of the P2 domain throughout MD simulations, the root mean square Fluctuation (RMSF) analysis was carried out on C-alpha atoms of each residue from the trajectory file. Radius of gyration (Rg) and solvent accessible area (SASA) of each protein were analyzed with the programs gmx gyrate and gmx sasa. Plots were visualized using Xmgrace tool and RMSF heat maps were generated using the Morpheus software (https://software.broadinstitute.org/morpheus/).

### 2.5. Molecular Docking

Norovirus GII.4 variants are capable of binding Lewis HBGA types [35]. To understand the detail of interactions between GII.4 capsid proteins and the receptor oligosaccharide, molecular docking of the capsid protein 3-D models and the HBGAs Lewis ^a^ trisaccharide and Lewis ^b^ tetrasaccharide, as the two main types of Lewis antigens, was performed using the COACH-D web server. The 3-D structures of the Lewis ^a^ and Lewis ^b^ were obtained from the PubChem database (https://pubchem.ncbi.nlm.nih.gov).

### 2.6. GII.4 Virus-Like Particle Production and Purification

Recombinant baculovirus expressing NoV GII.4 Sydney 2012 capsid protein was generated according to the instructions for the Bac-to-Bac Baculovirus Expression System (Thermo Fisher Scientific, Waltham, MA, USA). The virus-like particles (VLPs) were expressed in baculovirus-transformed Spodoptera frugiperda Sf9 insect cells, and then were purified on a step gradient of sucrose (15 to 60%) for 3 h at 112,700× *g*. The VLP-containing fractions were pooled, dialyzed, and stored at 4 °C until further use. Purified VLPs were characterized on Sodium dodecyl sulfate (SDS)-polyacryamide gel electrophoresis (PAGE), followed by Western blotting to confirm the presence of the capsid protein with an apparent molecular weight of 58 KDa. To ensure that the particles were intact, each preparation was examined by negative-stain electron microscopy (EM), using 1% ammonium molybdate. Protein concentration was determined by the Pierce BCA Protein Assay Kit (Thermo Fisher Scientific, Waltham, MA, USA).

### 2.7. Peptide Synthesis, Mouse Immunization, and HBGA Blocking Assay

There is evidence that the proteolytic degradation of short peptides can be markedly inhibited by N- and C-terminal residues modifications [36,37]. Thus, we had the antigenic epitopes A–E (predicted for GII.4 Sydney 2012) synthesized with N-terminal acetyl and C-terminal amide capping groups. Female BALB/c mice were injected intra-muscularly with three doses of the synthetic peptides three times (50 mg per mouse for each immunization) [38,39] on days 0, 14 and 28 in combination with cyclic di-adenosine monophosphate (c-di-AMP: 10 μg per mouse). Antibody concentration was measured in mouse sera collected two weeks after the last immunization with a quantitative ELISA [40]. Briefly, ELISA plates were coated with 1 μg NoV GII.4 VLPs at 4 °C overnight. A standard curve for quantitating IgG was generated with serial dilutions of mouse IgG in 1 × PBS (phosphate-buffered saline). After 3 times washing with 1 × PBS containing 0.05% Tween 20, the wells were blocked with 250 μL 5% non-fat milk in PBS for 2 h at room temperature (RT). Diluted mouse sera (1:1000 in PBS) were then added (100 μL) into the coated wells. After 1 h incubation at RT and washing (5×), the wells were treated with 100 μL of 1:2000 diluted (in 1 × PBS) horseradish peroxidase (HRP)-conjugated goat anti-mouse antibodies for 1 h at RT. After washing (5×), color was developed with 100 μL of TMB (3,3′,5,5′-tetramethylbenzidine) substrate solution (Sigma Aldrich, Taufkrichen, Germany) and absorbance values were measured at 450 nm on an Infinite F200 ELISA reader (Tecan, Crailsheim, Germany). Blocking assay using NoV GII.4 Sydney VLP and synthetic carbohydrate HBGA receptor H-type-3 was conducted as previously described [41] with some modifications. Microlitre plates were coated overnight at 4 °C with GII.4 VLP (400 ng/well). Serially diluted sera, starting from 1:100, were incubated with the coated VLPs at RT for 2 h. Then, biotinylated H-type-3 (Biosynth Carbosynth, Compton, UK) was added at a concentration of 20 µg/mL (100 µL/well). After 3 h incubation at 37 °C for 3 h, bound carbohydrates were detected with 1:3000 diluted streptavidin-conjugated HRP (Thermo Fisher Scientific, Waltham, MA, USA) and TMB substrate. Optical density (OD) value was measured at 450 nm, blocking index was calculated as the percentage of the maximum binding blocked by a certain serum dilution and was calculated as (1−(OD wells with serum/OD wells without serum) × 100%).

## 3. Results

### 3.1. Putative Liner B-Cell Epitopes Evolve Over Time

Linear B-cell epitopes were predicted on the basis of hydrophobic residues on NoV GII.4 capsid protein. As presented in Figure 1A, fifteen unique linear antigenic epitopes were predicted of which 10 discrete regions were identified as putative antigenic epitopes in the P domain, and five in the P2 domain cluster on the surface. Potential conformational epitope sites were also predicted for the surface-exposed region of the capsid. As shown in the graphical depiction of the surface-exposed epitopes in Figure 1B, the conformational epitopes coincide with the predicted linear epitopes. To figure out how these epitopes evolve over time, amino acid multiple alignments of GII.4 capsid protein consensus sequences spanning the years 1987 to 2016, and the sequence of the major outbreaks in 1987, 1997, 2002, 2004, 2006, 2009, and 2012 were generated. In comparing the sequences, 37 variable sites were detected within and/or at the neighboring sites of the P2 domain putative epitopes (Figure 1C). It also revealed that accumulated sequential amino acid mutations was correlated with increased prevalence of new emerging GII.4 variants, as shown in Figure 1C. The maximum likelihood ancestor counting analysis indicated that, in the P2 domain, amino acid residues 297, 373, 395, 412, and 413 within the epitopes evolved under positive selection.

**Figure 2:**
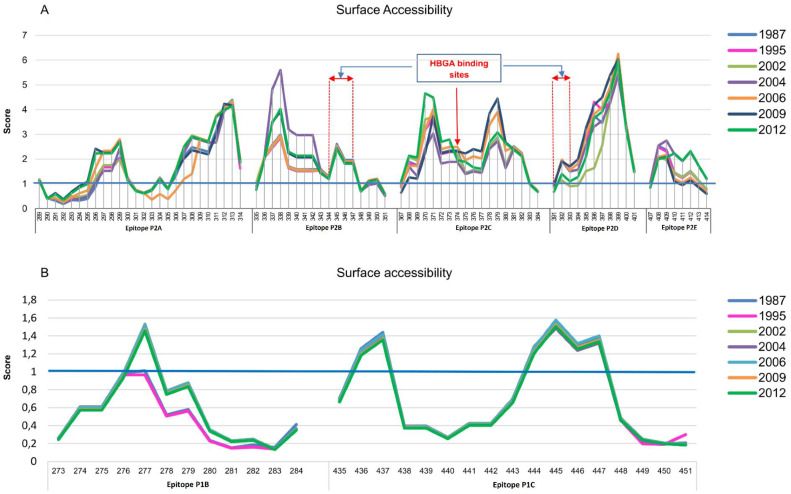
Surface accessibility of amino acid residues within epitopes sites. Capsid protein from norovirus variants responsible for outbreaks were assessed for changes in surface accessibility within epitopes sites using the Emini Surface Accessibility Prediction tool. (**A**) Epitopes within the P2 domain showed changes in a mutation-related manner. HBGA binding sites are located within epitopes P2B, P2C and P2D; (**B**) Epitope sites in the surface exposed sites of P1 domain (P1B and P1C) were stable over years.

Amino acid residues in P2 domain epitope sites were found to be differently surface accessible over years (Figure 2A). In contrast, no notable changes were detected for the surface exposed P1-domain epitopes P1B and P1C (Figure 2B). For epitope P2A, residues 296–299 and 307–311 were found to be flexible. However, the most accessible residues 312 and 313 stayed stable. In epitope P2B, residues 338–342 were found to be highly flexible. In contrast, at the neighboring sites 343, 344, 345, and 347 changes were restricted. In epitope 2C, residues 370, 371, 378, and 379, and in epitope P2D residues 396–399 showed highest surface accessibility. In epitope P2E, amino acids at positions 409–412 were shown to be flexible.

### 3.2. Amino Acid Substitutions Lead to Conformational Changes in Surface-Exposed Epitopes

**Figure 3:**
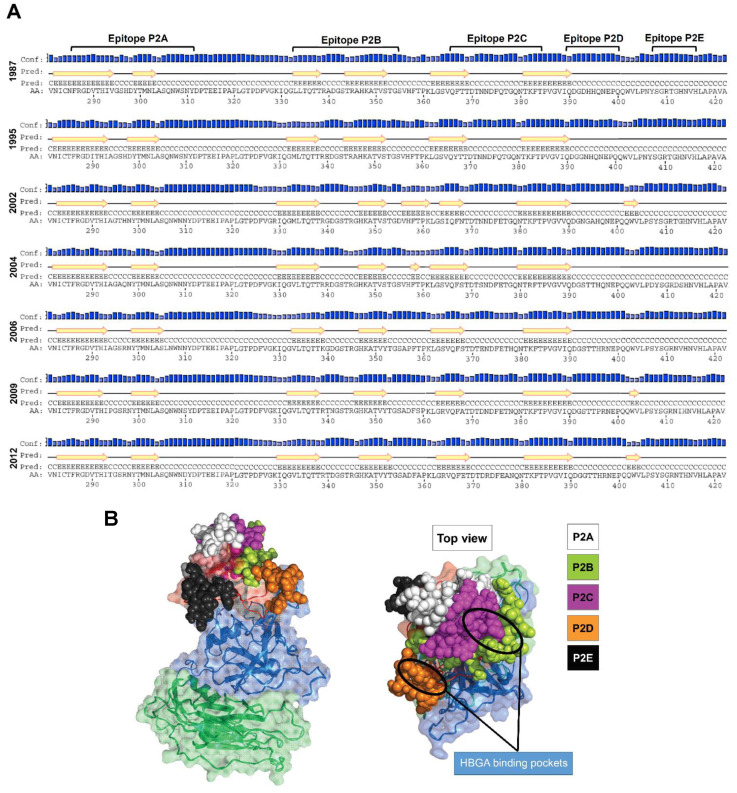
(**A**) Predicted secondary structure for norovirus GII.4 capsid protein. Yellow arrows represent β-strands. Amino acid substitution in variable sites caused notable changes in the P2 domain ß-strands. (**B**) Structural location of putative epitopes in GII.4 Sydney 2012 used in the present study.

Structural analysis of NoV GII.4 capsid proteins was conducted to find key amino acids affecting the conformation and accessibility of the putative epitopes. A secondary structure analysis revealed mutations in variable sites caused notable changes in the P2 domain ß-strands, as presented in Figure 3A. Early variants 1987 and 1995 showed similar secondary structures. However, in 2002, eight new amino acid substitutions at positions 346, 355, 365, 368, 376, 394, 395 and 407 resulted in changes in ß-strands within epitopes P2B and P2C, and insertion of two new ß-strands at positions 356–361 and 402–404. The new 356–361 ß-strand was replaced with a small ß-strand comprised of amino acids 358 and 359, in 2004, and later completely disappeared. In 2002, a ß-strand appeared between epitopes P2D and P2E regions (402–404), disappeared in 2004 and 2006/Minerva variants, and reappeared in 2009/New Orleans and 2012/Sydney.

We also analysis the 3-D homology models of the GII.4 norovirus proteins. For this aim, 3-D homology models were generated. Structural alignment between the models and real capsid protein crystal structure revealed that all modelled protein structures have RMSD < 2.0 A indicating that all the models were in the range of native conformation. The modelled protein structures were energy minimized using Modrefiner and steric clashes were removed using Chiron tools (Department of Biochemistry and Molecular Biology, Penn State Neuroscience Institute, Hershey, PA, USA). After refinement of each modelled protein structures were validated using ProSA-web server. A Z-score of −6 to −6.44 was measured for the modelled proteins confirming that they are in the range of native conformations (Figure S1). Protein geometry analysis of each model was evaluated using Ramachandran plot (see methods) this indicated that all models have > 90% favored regions (Supplementary Figure S2). The predicted epitopes were mapped on the surface of the capsid protein, where HBGA binding pockets share amino acid sites within epitopes P2B, P2C, and P2D. Figure 3B represents the structure location of the putative epitopes in GII.4 Sydney 2012. Comparing the 3-D models, we observed notable changes in location and conformation of the surface-exposed epitopes (Supplementary Figure S3).

### 3.3. MD Simulation Revealed Conformational Flexcibilty in the Putative Epitope Sites over Time

**Figure 4:**
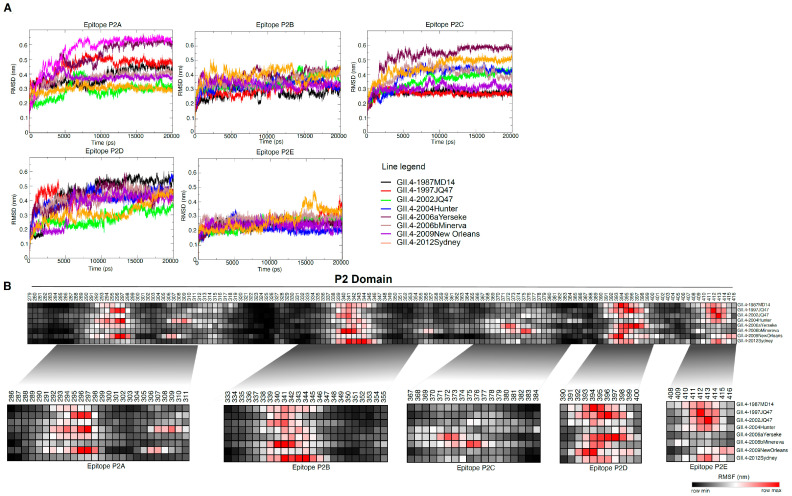
Molecular dynamic simulation analysis of NoV GII.4 P2 domain. Homology 3-D models were generated, protein models were visualized and after validation analysis were subjected to MD simulation. (**A**) RMSD analysis showed distinct patterns of instability in the epitopes sites within the P2 domain of GII.4 variants. (**B**) RMSF analysis revealed high fluctuation in epitope sites. Black and red color denotes low and high RMSF values.

To delineate protein motions at the atomic level, we conducted molecular dynamics (MD) simulation. After 20 ns of simulation, analysis of the RMSD, RMSF, Rg and SASA parameters revealed changes in the P2 domain antigenic epitopes at molecular level over the years. RMSD is an indicator of structural stability, which is widely used for evaluating the structural differences between proteins. The average RMSD of the structures ranged from 0.3 to 0.5 nm during trajectory. A comparison of RMSD values revealed distinct patterns of instability in the epitope sites in different years (Figure 4A). We performed RMSF analysis to understand contribution of each residue to the flexibility of the epitopes throughout the MD simulations. As shown in Figure 4B, amino acids within predicted epitopes contributed most to flexibility. Epitope P2A was highly flexible, particularly amino acids 296–299 and 308–311. Whereas amino acids 343–347 in epitope P2B were conformationally stable, 337–341 were highly flexible in most of the variants over the time. Amino acid residues 372, 373, 375 and 376 (Epitope P2C) and residues 391–399 (Epitope P2D) as well as 412 and 413 (Epitope P2E) were also highly flexible. In currently circulating Sydney 2012 and 2015 variants epitopes P2A and P2D were found to be more stable than in the earlier GII.4 variants. It seems that T294 substitution in epitope P2A, and new E268H, R373N, and A377T mutations in epitope P2D resulted in increased protein rigidity, which can decrease antibody binding. Rg analysis is mostly used to determine protein compactness [42]. This varied for the predicted epitopes over the years. Generally, epitopes P2C, P2D and P2E were more compact than P2A and P2B. In agreement with the results of the surface accessibility prediction (Figure 2), calculation of the accessibility surface area showed that solvent accessibility of epitopes varied throughout the years. No substantial deviations were observed in the HBGA binding region (residues 343–348) in the epitope P2B across the years. Taken together, our MD simulation revealed dynamic changes of epitopes throughout years.

### 3.4. Amino Acid Substitutions in Epitopes P2B, P2C and P2D Affect HBGA Binding

**Figure 5:**
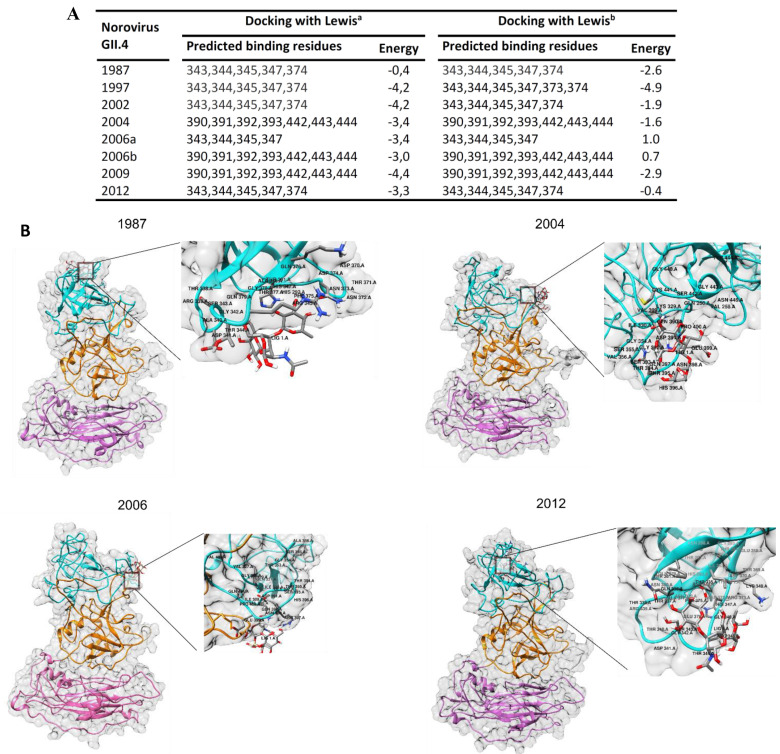
Molecular docking of NoV capsid protein and HBGA. (**A**) Predicted HBGA binding sites. Comparing GII.4 variants, two HBGA binding patterns were predicted; each GII.4 variant showed similar binding pattern for the both Lewis^a^ and Lewis^b^ saccharides. (**B**) 3-D protein structure models of GII.4 capsid proteins from 1987, 2004, 2006 and 2012 are represented along docked pose (zoomed plot) of HBGA with P2 domains.

Molecular docking of the reconstructed models to the HBGA oligosaccharides was conducted to understand the effect of amino acid substitutions in surface-exposed epitopes on HBGA binding. The data revealed changes in binding pattern and binding affinity to HBGA carbohydrate (Figure 5) as a result of mutations in the epitope sites, although HBGA binding sites were conserved during the years. The binding pocket, in the 1987, 1997 and 2002 was found to share a part of the epitope P2B region (aa 343–347) and a site within epitope P2C (aa374). Following amino acid substitution at positions 340 (A340E) and 367 (F367Y) in the 1995/Houston variant, a noticeable increase in binding affinity was determined when compared to the early 1987 variant. It seems that mutations at positions 340 (A340E) and 367 (F367Y) cause an increase in binding affinity in 1997 variants compared to the 1987 variant. Later, in 2004/Hunter, binding sites have shift to the second binding pocket which shared amino acid residues 390 to 393 in epitope P2D and residues 442 to 443 from a surface-exposed region in P1 domain. Followed by amino acid substitutions within epitopes P2B (S359A) and P2C (A368E, N373R, T377A) in Sydney 2012, binding sites again shift to the first binding pocket within epitopes P2B and P2C. More details regarding the molecular docking results are shown in Figure 5. Overall, this analysis pinpointed the key residues within putative epitopes interacting with HBGA.

### 3.5. Epitopes P2B, P2C and P2D Elicited Blocking Antibodies

**Figure 6:**
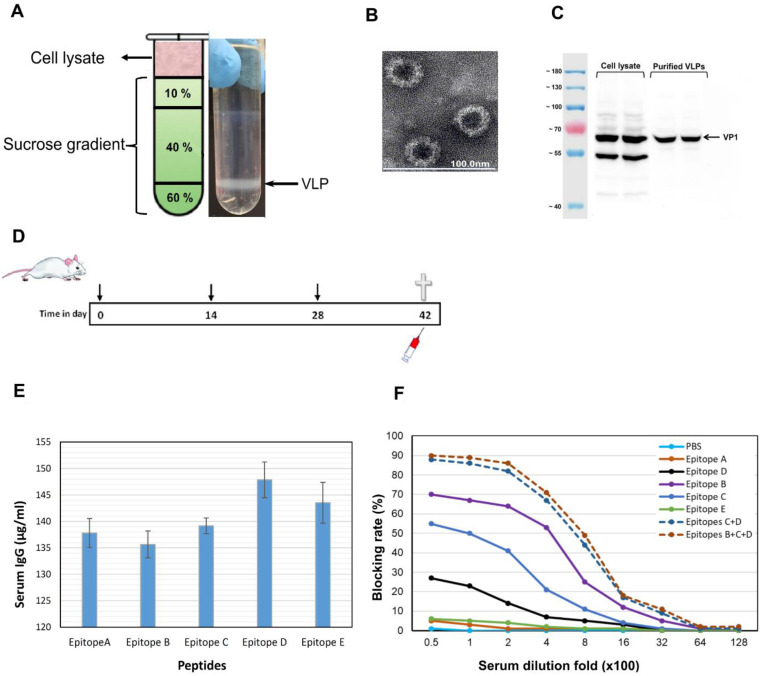
Characterization of predicted epitopes P2A-E within NoV GII.4 capsid protein. NoV GII.4 Sydney 2012 VLPs were produced using baculovirus expression system: (**A**) NoV VLPs were purified on sucrose gradient; (**B**) negative-stained VLPs were visualized by electron microscopy; (**C**) VLPs were tested with Western blotting. (**D**) BALB/c mice were immunized with synthetic epitopes three times; two weeks after the last immunization, serum antibody level was determined by ELISA (**E**) and blockade of NoV GII.4 Sydney 2012 VLPs binding to HBGA H-type-3 receptor by sera of the immunized mice was tested (**F**): Blocking activities were determined for pooled sera from five mice in each group. PBS served as negative control.

NoV GII.4 VLP were generated in ExpSf9 insect cells using the BAC to BAC baculovirus expression system and then were purified on sucrose gradient (Figure 6A); the production of VLPs were confirmed by electron microscopy (Figure 6B) and Western blotting (Figure 6C). To study the immunogenicity of the predicted epitopes P2A-E, we measured blocking antibodies elicited in wild-type mice by synthetic epitope peptides. As shown in Figure 6E, we found that epitopes P2B, P2C and P2D elicited blocking antibodies with the blocking rate of maximum 68%, 55%, and 28%, respectively against GII.4 Sydney 2012 (Figure 6F). In contrast, sera of the mice immunized with epitopes P2A and P2E showed low blocking rate of less than 7%. Blocking rate was found to increase to 80% when we used a pooled serum of epitopes P2B and P2C antisera for the assay. With including epitope P2D antisera in the serum pool, a slight increase in the blocking activity was detected.

## 4. Discussion

Norovirus GII.4 genotype exhibits epochal evolution, whereby major circulating strains give rise to emergent strains, likely due to escape from herd immunity [9,43]. It appears that the GII.4 genotype evolved linearly over time with bursts of adaptation that gave rise to new variants emerging from previous circulating variants, often via recycling of residues within important surface-exposed epitopes. Periodic increases in amino acid motif diversity at these antigenic epitopes coincided with periods of high NoV activity. Some of these changes are likely to be phenotypically neutral, as they do not affect surface areas. Diversity at epitope sites is associated with maintaining sufficient antigenic diversity within the virus population allowing for the persistence of GII.4 variants in the pre-exposed human population between epidemic waves. This therefore suggests that these sites contribute to immune evasion and hence the persistence of GII.4 variants in the population.

Prior to this work, a number of discreet amino acids, in particular residues 296–298, 393–395 and also surface residues at 333, 340, 356, 368, 372, 407, and 412–413 have been described as potential evolving GII.4 antibody epitopes which change between GII.4 variants [9,10,11,14,16]. These amino acids tend to cluster on loops and ridges of the P2 domain where they would be more accessible for antibody interaction. Beyond bioinformatics predictions, only a few experimental studies have shown empirical evidence mapping GII.4 antigenic change. Allen and colleagues [14] compared the reactivity of five monoclonal antibodies against a pre- and post-2002 epidemic GII.4 strain, and identified conformational epitopes composed of residues 294–296 and 393–395. In a study using a panel of mouse monoclonal antibodies (MAbs) against GII.4.2006 and GII.4.2009 virus-like particles (VLPs), Lindesmith et al. [44] identified residues 294, 296, 297, 298, 368, and 372 as important antigenic sites in these strains. In another study, they also described two evolving GII.4-specific blockade epitopes A (294–298 and 368–372) and D (393–395) by comparing the reactivity of human anti-GII.4 mAbs to GII.4 VLPs [8].

In present study, we predicted five linear antigenic epitopes (P2A-E) in the P2 domain with surface-exposed areas which can potentially be used for improvement of NoV GII.4 vaccines. We propose a model of antigenic evolution for GII-4 NoV strains based on sequence diversity, structural modelling and dynamic evolution of these surface-exposed antigenic regions over time. They accumulated sequential amino acid mutations that correlated with increased prevalence of emerging GII.4 variants which sequentially change suggesting that these sites may play role in the escape from herd immunity. These sites undergo selective amino acid changes that coincide with the appearance of new epidemiologically significant GII.4 variants in the population. Evidence supporting this includes greater positive selection pressure on the amino acids in the P2 domain than in the remaining capsid protein. Although it is hard to determine how changes in amino acids will contribute to the specific antigenicity without further laboratory investigation, such changes will undoubtedly affect recognition between antibody and epitopes. We explored the value of modelling the predicted surface area derived from the different amino acid substitutions in variable sites as predictors of significant structural changes correlated with the emergence and/or switch of epidemic strains. This analysis revealed marked conformational changes in the P2 subdomain with major surface area motifs.

Analysis of surface accessibility revealed that the predicted P2 domain epitopes expose to the surface at varying threshold over time. For antibody recognition and binding, the antigenic sites must be surface accessible and flexibility in these sites allows the formation of an antigen-antibody interface since flexible regions can adjust their conformation upon interaction with an antibody. Therefore, flexibility of a region is an indicator of the existence of a potential antigenic site [34]. Positions 296–299 and 308–311 were recognized as the most flexible sites in epitope P2A, suggesting as antigenic residues for this epitope. In epitope P2B, surface accessibility changes at positions 343, 344, 345, and 347, which are involved in HBGA binding [14,45], was restricted. However, neighboring sites of epitope P2B (residues 338 to 442) were highly flexible. It seems that amino acid residues at HBGA binding site in epitope P2B are under pressure to maintain receptor-binding functionality even when epitope antigenicity changes. Residue 374 in epitope P2C that also binds to HBGA was differently exposed on the capsid protein surface over time by mutations at neighboring residues. It suggests that conformational changes in this region may play important role in defining binding pattern to HBGA.

The binding specificity of the norovirus capsid protein to different HBGAs differs among the genotypes and genogroups [43], leading to genotypic-susceptibility [46]. It has been proposed that HuNoVs are under selective pressure for binding to a wide diversity of HBGA containing receptors in the gut. Here we compare HBGA binding of the GII.4 variants by molecular docking of reconstructed capsid protein structures and the HBGA oligosaccharides Lewis ^a^ and Lewis ^b^. The results revealed two binding pockets for interaction of the capsid protein with HBGA. In 1987, 1995, and 2002 variants, the HBGA binding pocket shares a part of epitope P2B (aa 343, 344, 345, and 347) and a residue 374 in epitope P2C. It seems that mutations at positions 340 and 367 caused a clear increase in binding affinity in 1995 variant compared to the 1987. Later, in 2004/Hunter, 2006/Minerva and 2009/New Orleans, due to mutations in epitope P2B (aa 340, 346, and 355) and epitope P2C (aa 368 and 376) and subsequent conformational changes in the binding region, binding sites have shift to an alternative binding pocket which shares residues 391–393 in epitope P2D and residues 442, 443 and 444 in the surface-exposed P1C epitope within the P1 domain. Mutations in neighboring sites of the binding pocket within epitope P2C and P2D may likely change the number of H-bonds through residue-residue approximation constraints with adjacent amino acids. In Sydney 2012, similar binding pocket as early variants 1987–2002 was predicted. It can be due to a notable decrease in the accessibility of HBGA binding sites 391–393 in epitope P2D, and significant conformational changes in epitope P2C as a result of three new substitutions A368E, N373R, and T377A. Interestingly, in Sydney 2012 and 2015 variants, the flexibility of the protein was considerably reduced in P2A and P2D. It seems that T294 substitution in epitope P2A, and new E268H, R373N and A377T mutations in epitope P2D resulted in increased protein rigidity which can make the epitopes unsuitable for interacting with antibodies. Robust data exhibit altered antigenicity and HBGA binding over time in VLPs of major outbreak and pandemic GII.4 strains, and identify strain-specific potentially neutralizing antibodies in GII.4 NoVs [45]. Similar to HuNoV GII.4, in influenza A virus amino acid substitutions in hemagglutinin (HA) 1 domain of the HA gene drive antigenic variation leading to escape from existing herd immunity [47,48]. Interestingly, both NoV GII.4 P2 domain (Figure 1C) and influenza HA1 domain [47], which interact with antibodies and binding ligands, contain many amino acids undergoing positive selection. We observed periodic increases in amino acid motif diversity at antigenic sites coincided with periods of high NoV activity. We predict that these sites are part of major epitopes on the virus surface, and that diversity at these sites is associated with maintaining sufficient antigenic diversity within the virus population allowing for the persistence of GII.4 variants in the pre-exposed human population between epidemic waves. This therefore suggests that these sites contribute to immune evasion and hence the persistence of GII.4 variants in the population.

HBGA blocking ability is suggested to be an important factor in achieving protective immunity against NoV (19,52) as neutralizing antibodies raised against NoV have been shown to block the binding of NoV VLPs to the synthetic HBGA receptor [49,50,51]. For this reason, the present vaccine approaches are focused on stimulating heterotypic HBGA blocking antibodies [52,53]. In this work, the carbohydrate blockade potential of antibodies induced by the synthetic putative epitopes was studied. The results demonstrated that the epitopes P2B and P2C antisera were able to block the GII.4 Sydney 2012 VLP binding to the HBGA receptor with the blocking rate of 68% and 55%, respectively. This was in agreement with the results of docking prediction (Figure 5) in which HBGA binding pocket in GII.4 Sydney 2012 was predicted to share amino acids sites within epitopes P2B and P2C. Due to the low binding affinity of HBGA to the binding sites within epitope P2D (data not shown), low blocking activity was measured for epitope P2D antisera. Interestingly, blocking rate was shown to increase to 80% using the mixed sera of epitopes P2B, P2C and P2D. The location of three epitopes on the surface-exposed part of the capsid and directly proximal to the HBGA binding pockets, suggesting that amino acid substitutions in these regions may work in concert to change the structure of epitopes resulting in receptor switching and also escape from neutralizing antibodies in new emerging variants [9,16,45,54]. In contrast to epitopes P2B, P2C, and P2D, we could not detect a significant level of blocking antibodies for the epitopes P2A and P2E antisera. For these two epitopes, the patterns of amino acid substitutes at the variable residues suggest that they may play role in the evolution of new variants by influence on the physiochemical properties of the amino acid replacements in the neighbour epitopes.

**Figure 7:**
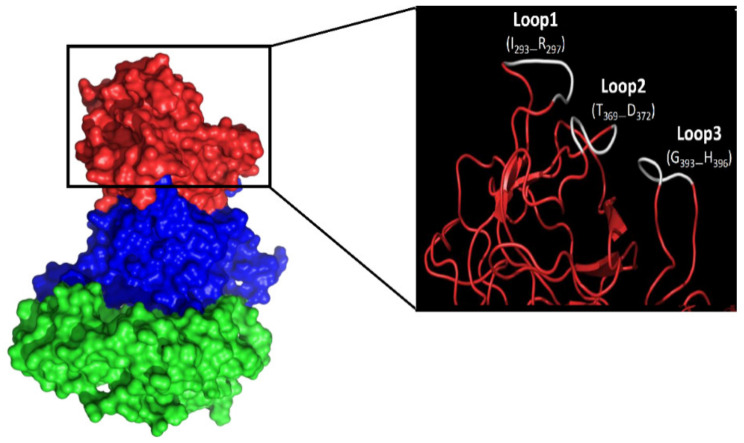
Norovirus capsid protein surface loops. Red indicates P2 domain, while blue and green indicate P1 and S domain. The surface loops that are suitable sites for insertion of foreign antigen are shown in white.

These data demonstrate the potentials of the identified linear epitopes P2B, P2C and P2D as blockade epitopes in generation of chimeric GII.4 VLPs, polyvalent synthetic and/or DNA-based peptide vaccines against NoV infection. Here, based on our findings, we suggest generating chimeric GII.4 VLPs harboring blocked epitopes from GII.4 variants with different HBGA binding pattern predicted with molecular docking. The use of such vaccines will allow broad coverage of the target NoV GII.4 variants as the most prevalent genotype in outbreaks. Previous studies indicated three loops (Loop1: 293–297; Loop2: 371–372; Loop3: 391–394) on the surface part of the NoV P particles [55], which can tolerate large sequence insertions [55,56]. Comparing the 3-D homology models of the GII.4 variants, we also identified similar loops on the surface of the capsid protein (Figure 7). These loops are located on the outermost surface of the predicted epitopes P2A (Loop1: 293–297), P2C (Loop2: 369–372), and P2D (Loop3: 393–396), suggesting that they are potential sites for surface displaying of foreign antigens. We have shown that these loops can tolerate large sequence insertions (unpublished data).

## 5. Conclusions

In this work, we investigated surface-exposed linear antigenic epitope within NoV GII.4 capsid protein at sequence and structural level. Along with bioinformatics analysis, including molecular dynamic stimulation and molecular docking, the characteristic HBGA blocking rates in mice demonstrates the potential of the three identified linear epitopes P2B, P2C and P2D as blockade epitopes in generation broad reactive vaccines against NoV GII.4. The use of such vaccines will likely reduce the challenges associated with vaccine development against norovirus and allow broad coverage of new emergent variants.

## Data Availability

No new data were created or analyzed in this study. Data sharing is not applicable to this article.

## Supplementary Materials

The following are available online at https://www.mdpi.com/2076-393X/9/1/52/s1, Figure S1: Scatter plots depict the modelled GII.4.VP1 structure validation by ProSA-web algorithm. The black dots reveal that all modeled structures fall within the series of scores found on related sized proteins with an X-ray quality, Figure S2: The Ramachandran plots of GII.4.VP1 protein structure models from different years, Figure S3: Three-dimensional surface plots depict the GII.4.VP1 structures color coded according to domain architecture (left side) and epitopes in P2 domain (right side).

## References

[1] Hall A.J., Glass R.I., Parashar U.D. (2016). New insights into the global burden of noroviruses and opportunities for prevention. Expert Rev. Vaccine..

[2] Havelaar A.H., Kirk M.D., Torgerson P.R., Gibb H.J., Hald T., Lake R.J., Praet N., Bellinger D.C., De Silva N.R., Gargouri N. (2015). World Health Organization Global estimates and regional comparisons of the burden of foodborne disease in 2010. PloS Med..

[3] Bartsch S.M., Lopman B.A., Ozawa S., Hall A.J., Lee B.Y. (2016). Global economic burden of norovirus gastroenteritis. PLoS ONE.

[4] Lopman B.A., Steele D., Kirkwood C.D., Parashar U.D. (2016). The vast and varied global burden of norovirus: Prospects for prevention and control. PloS Med..

[5] Vinjé J. (2015). Advances in laboratory methods for detection and typing of norovirus. J. Clin. Microbiol..

[6] Kroneman A., Vega E., Vennema H., Vinjé J., White P.A., Hansman G., Green K., Martella V., Katayama K., Koopmans M. (2013). Proposal for a unified norovirus nomenclature and genotyping. Arch. Virol..

[7] Chhabra P., de Graaf M., Parra G.I., Chan M.C.-W., Green K., Martella V., Wang Q., White P.A., Katayama K., Vennema H. (2019). Updated classification of norovirus genogroups and genotypes. J. Gen. Virol..

[8] Lindesmith L.C., Costantini V., Swanstrom J., Debbink K., Donaldson E.F., Vinjé J., Baric R.S. (2013). Emergence of a norovirus GII. 4 strain correlates with changes in evolving blockade epitopes. J. Virol..

[9] Lindesmith L.C., Donaldson E.F., LoBue A.D., Cannon J.L., Zheng D.-P., Vinje J., Baric R.S. (2008). Mechanisms of GII. 4 norovirus persistence in human populations. PloS Med.

[10] Bull R.A., Eden J.-S., Rawlinson W.D., White P.A. (2010). Rapid evolution of pandemic noroviruses of the GII. 4 lineage. PloS Pathog..

[11] Allen D.J., Gray J.J., Gallimore C.I., Xerry J., Iturriza-Gómara M. (2008). Analysis of amino acid variation in the P2 domain of the GII-4 norovirus VP1 protein reveals putative variant-specific epitopes. PLoS ONE.

[12] Bok K., Abente E.J., Realpe-Quintero M., Mitra T., Sosnovtsev S.V., Kapikian A.Z., Green K.Y. (2009). Evolutionary dynamics of GII. 4 noroviruses over a 34-year period. J. Virol..

[13] Lindesmith L.C., Donaldson E.F., Baric R.S. (2011). Norovirus GII. 4 strain antigenic variation. J. Virol..

[14] Allen D.J., Noad R., Samuel D., Gray J.J., Roy P., Iturriza-Gómara M. (2009). Characterisation of a GII-4 norovirus variant-specific surface-exposed site involved in antibody binding. Virol. J..

[15] Zakikhany K., Allen D.J., Brown D., Iturriza-Gómara M. (2012). Molecular evolution of GII-4 Norovirus strains. PLoS. ONE..

[16] Donaldson E.F., Lindesmith L.C., Lobue A.D., Baric R.S. (2008). Norovirus pathogenesis: Mechanisms of persistence and immune evasion in human populations. Immunol. Rev..

[17] Klepeis J.L., Lindorff-Larsen K., Dror R.O., Shaw D.E. (2009). Long-timescale molecular dynamics simulations of protein structure and function. Curr. Opin. Struct. Biol..

[18] Kumar S., Stecher G., Tamura K. (2016). MEGA7: Molecular evolutionary genetics analysis version 7.0 for bigger datasets. Mol. Biol. Evol..

[19] EL-Manzalawy Y., Dobbs D., Honavar V. (2008). Predicting linear B-cell epitopes using string kernels. J. Mol. Recognit..

[20] Kobayashi M., Matsushima Y., Motoya T., Sakon N., Shigemoto N., Okamoto-Nakagawa R., Nishimura K., Yamashita Y., Kuroda M., Saruki N. (2016). Molecular evolution of the capsid gene in human norovirus genogroup II. Sci. Rep..

[21] Sun J., Wu D., Xu T., Wang X., Xu X., Tao L., Li Y., Cao Z.-W. (2009). SEPPA: A computational server for spatial epitope prediction of protein antigens. Nucleic Acids Res..

[22] Ponomarenko J., Bui H.-H., Li W., Fusseder N., Bourne P.E., Sette A., Peters B. (2008). ElliPro: A new structure-based tool for the prediction of antibody epitopes. BMC Bioinform..

[23] Kearse M., Moir R., Wilson A., Stones-Havas S., Cheung M., Sturrock S., Buxton S., Cooper A., Markowitz S., Duran C. (2012). Geneious Basic: An integrated and extendable desktop software platform for the organization and analysis of sequence data. Bioinformatics.

[24] Kim Y., Ponomarenko J., Zhu Z., Tamang D., Wang P., Greenbaum J., Lundegaard C., Sette A., Lund O., Bourne P.E. (2012). Immune epitope database analysis resource. Nucleic Acids Res..

[25] Kolaskar A., Tongaonkar P.C. (1990). A semi-empirical method for prediction of antigenic determinants on protein antigens. Febs Lett..

[26] Emini E.A., Hughes J.V., Perlow D., Boger J. (1985). Induction of hepatitis A virus-neutralizing antibody by a virus-specific synthetic peptide. J. Virol..

[27] Karplus P., Schulz G. (1985). Prediction of chain flexibility in proteins. Naturwissenschaften.

[28] Drozdetskiy A., Cole C., Procter J., Barton G.J. (2015). JPred4: A protein secondary structure prediction server. Nucleic Acids Res..

[29] McGuffin L.J., Bryson K., Jones D.T. (2000). The PSIPRED protein structure prediction server. Bioinformatics.

[30] Yang J., Yan R., Roy A., Xu D., Poisson J., Zhang Y. (2015). The I-TASSER Suite: Protein structure and function prediction. Nat. Methods.

[31] Pettersen E.F., Goddard T.D., Huang C.C., Couch G.S., Greenblatt D.M., Meng E.C., Ferrin T.E. (2004). UCSF Chimera—a visualization system for exploratory research and analysis. J. Comput. Chem..

[32] Wiederstein M., Sippl M.J. (2007). ProSA-web: Interactive web service for the recognition of errors in three-dimensional structures of proteins. Nucleic Acids Res..

[33] Pronk S., Páll S., Schulz R., Larsson P., Bjelkmar P., Apostolov R., Shirts M.R., Smith J.C., Kasson P.M., Van Der Spoel D. (2013). GROMACS 4.5: A high-throughput and highly parallel open source molecular simulation toolkit. Bioinformatics.

[34] Mian I.S., Bradwell A.R., Olson A.J. (1991). Structure, function and properties of antibody binding sites. J. Mol. Biol..

[35] Huang P., Farkas T., Marionneau S., Zhong W., Ruvoën-Clouet N., Morrow A.L., Altaye M., Pickering L.K., Newburg D.S., LePendu J. (2003). Noroviruses bind to human ABO, Lewis, and secretor histo-blood group antigens: Identification of 4 distinct strain-specific patterns. J. Infect. Dis..

[36] Brinckerhoff L.H., Kalashnikov V.V., Thompson L.W., Yamshchikov G.V., Pierce R.A., Galavotti H.S., Engelhard V.H., Slingluff C.L. (1999). Terminal modifications inhibit proteolytic degradation of an immunogenic mart-127–35 peptide: Implications for peptide vaccines. Int. J. Cancer.

[37] Powell M.F., Grey H., Gaeta F., Sette A., Colón S. (1992). Peptide stability in drug development: A comparison of peptide reactivity in different biological media. J. Pharm. Sci..

[38] Belyakov I.M., Derby M.A., Ahlers J.D., Kelsall B.L., Earl P., Moss B., Strober W., Berzofsky J.A. (1998). Mucosal immunization with HIV-1 peptide vaccine induces mucosal and systemic cytotoxic T lymphocytes and protective immunity in mice against intrarectal recombinant HIV-vaccinia challenge. Proc. Natl. Acad. Sci..

[39] Ahlers J.D., Takeshita T., Pendleton C.D., Berzofsky J.A. (1997). Enhanced immunogenicity of HIV-1 vaccine construct by modification of the native peptide sequence. Proc. Natl. Acad. Sci..

[40] Afridi S.Q., Moeini H., Kalali B., Wettengel J.M., Quitt O., Semper R., Gerhard M., Protzer U., Hoffmann D. (2019). Quantitation of norovirus-specific IgG before and after infection in immunocompromised patients. Braz. J. Microbiol..

[41] Tamminen K., Huhti L., Koho T., Lappalainen S., Hytönen V.P., Vesikari T., Blazevic V. (2012). A comparison of immunogenicity of norovirus GII-4 virus-like particles and P-particles. Immunology.

[42] Lobanov M.Y., Bogatyreva N., Galzitskaya O. (2008). Radius of gyration as an indicator of protein structure compactness. Mol. Biol..

[43] Donaldson E.F., Lindesmith L.C., LoBue A.D., Baric R.S. (2010). Viral shape-shifting: Norovirus evasion of the human immune system. Nat. Reviews. Microbiol..

[44] Lindesmith L.C., Debbink K., Swanstrom J., Vinjé J., Costantini V., Baric R.S., Donaldson E.F. (2012). Monoclonal antibody-based antigenic mapping of norovirus GII. 4-2002. J. Virol..

[45] Debbink K., Donaldson E.F., Lindesmith L.C., Baric R.S. (2012). Genetic mapping of a highly variable norovirus GII. 4 blockade epitope: Potential role in escape from human herd immunity. J. Virol..

[46] Lindesmith L., Moe C., Marionneau S., Ruvoen N., Jiang X., Lindblad L., Stewart P., LePendu J., Baric R. (2003). Human susceptibility and resistance to Norwalk virus infection. Nat. Med..

[47] Koelle K., Cobey S., Grenfell B., Pascual M. (2006). Epochal evolution shapes the phylodynamics of interpandemic influenza A (H3N2) in humans. Science.

[48] Koelle K., Ratmann O., Rasmussen D.A., Pasour V., Mattingly J. (2011). A dimensionless number for understanding the evolutionary dynamics of antigenically variable RNA viruses. Proc. R. Soc. B: Biol. Sci..

[49] Harrington P.R., Lindesmith L., Yount B., Moe C.L., Baric R.S. (2002). Binding of Norwalk virus-like particles to ABH histo-blood group antigens is blocked by antisera from infected human volunteers or experimentally vaccinated mice. J. Virol..

[50] Cannon J.L., Lindesmith L.C., Donaldson E.F., Saxe L., Baric R.S., Vinjé J. (2009). Herd immunity to GII. 4 noroviruses is supported by outbreak patient sera. J. Virol..

[51] Tan M., Fang P., Chachiyo T., Xia M., Huang P., Fang Z., Jiang W., Jiang X. (2008). Noroviral P particle: Structure, function and applications in virus–host interaction. Virology.

[52] LoBue A.D., Lindesmith L., Yount B., Harrington P.R., Thompson J.M., Johnston R.E., Moe C.L., Baric R.S. (2006). Multivalent norovirus vaccines induce strong mucosal and systemic blocking antibodies against multiple strains. Vaccine.

[53] Herbst-Kralovetz M., Mason H.S., Chen Q. (2010). Norwalk virus-like particles as vaccines. Expert Rev. Vaccines.

[54] Shanker S., Choi J., Sankaran B., Atmar R., Estes M., Prasad B. (2011). Structural Analysis of HBGA Binding Specificity in a Norovirus GII. 4 Epidemic Variant: 632 Implications for Epochal Evolution. J. Virol..

[55] Cao S., Lou Z., Tan M., Chen Y., Liu Y., Zhang Z., Zhang X.C., Jiang X., Li X., Rao Z. (2007). Structural basis for the recognition of blood group trisaccharides by norovirus. J. Virol..

[56] Tan M., Huang P., Xia M., Fang P.-A., Zhong W., McNeal M., Wei C., Jiang W., Jiang X. (2011). Norovirus P particle, a novel platform for vaccine development and antibody production. J. Virol..

