# Supplementary material for: Linear B-Cell Epitopes in Human Norovirus GII.4 Capsid Protein Elicit Blockade Antibodies
Source: Vaccines (Basel). 2021 Jan 14;9(1):52. doi: 10.3390/vaccines9010052 (PMC7830539; doi:10.3390/vaccines9010052)
Supplement: Supplementary file 1 [file vaccines-09-00052-s001.pdf]

Supplementary Figure S1

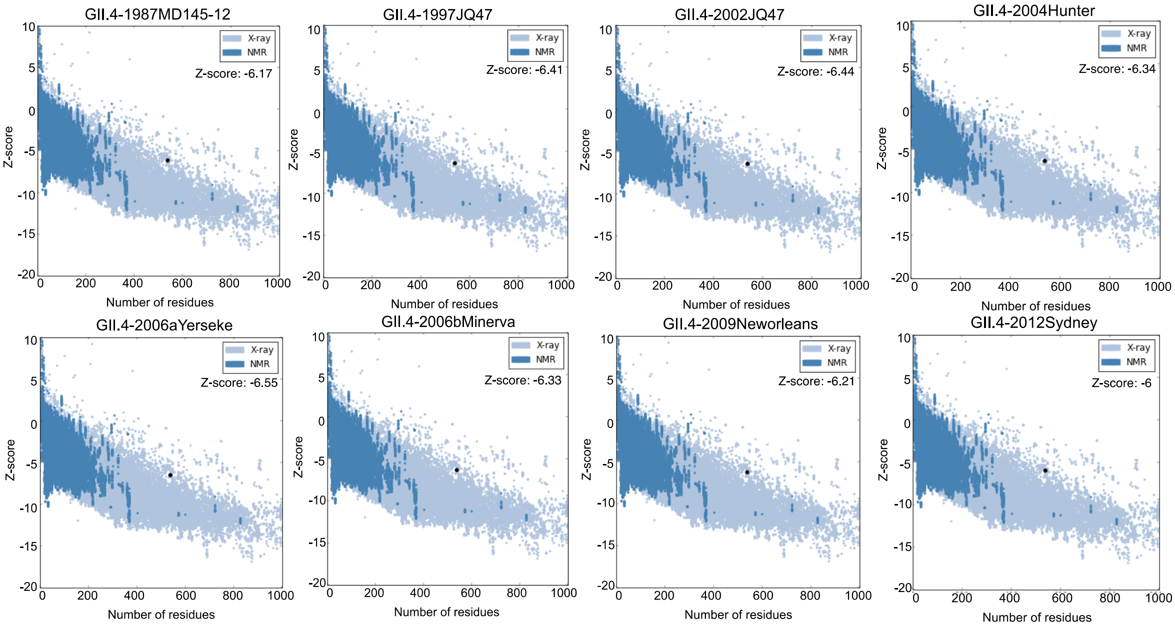

Figure S1. Scatter plots depict the modelled GII.4.VP1 structure validation by ProSA-web algorithm. The black dots reveals that all modeled structures falls within the series of scores found on related sized proteins with an X-ray quality.

Supplementary Figure S2

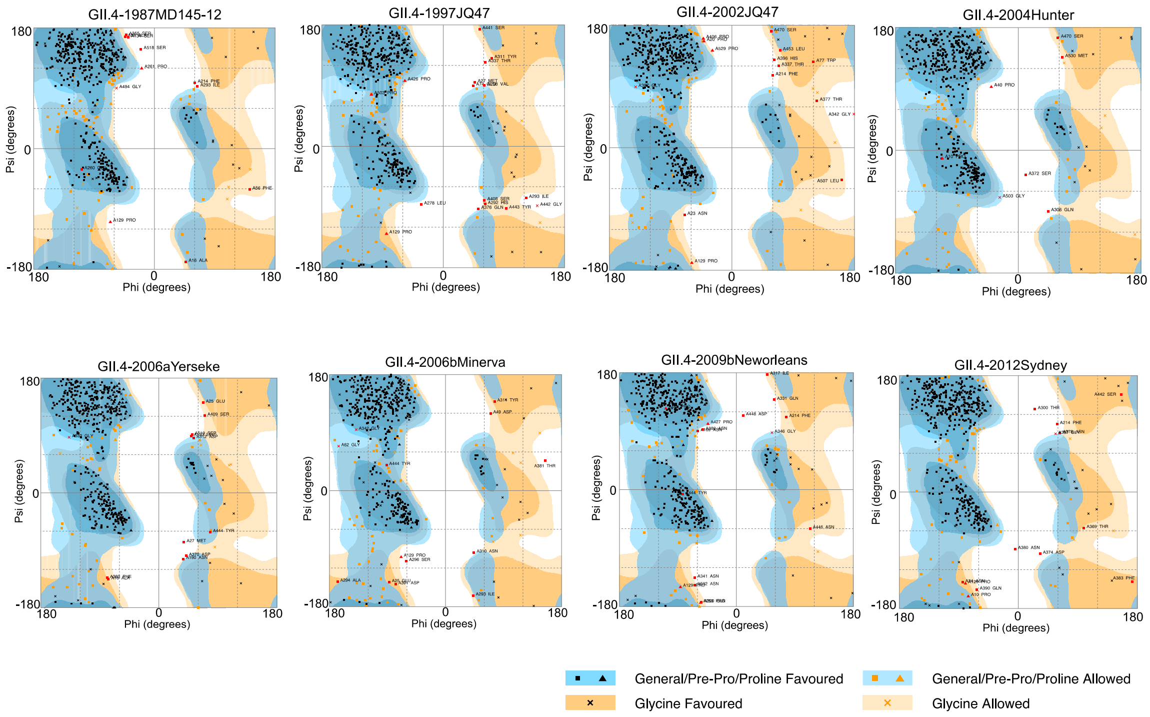

Figure S2. The Ramachandran plots of GII.4.VP1 protein structure models from different years.

Supplementary Figure S3

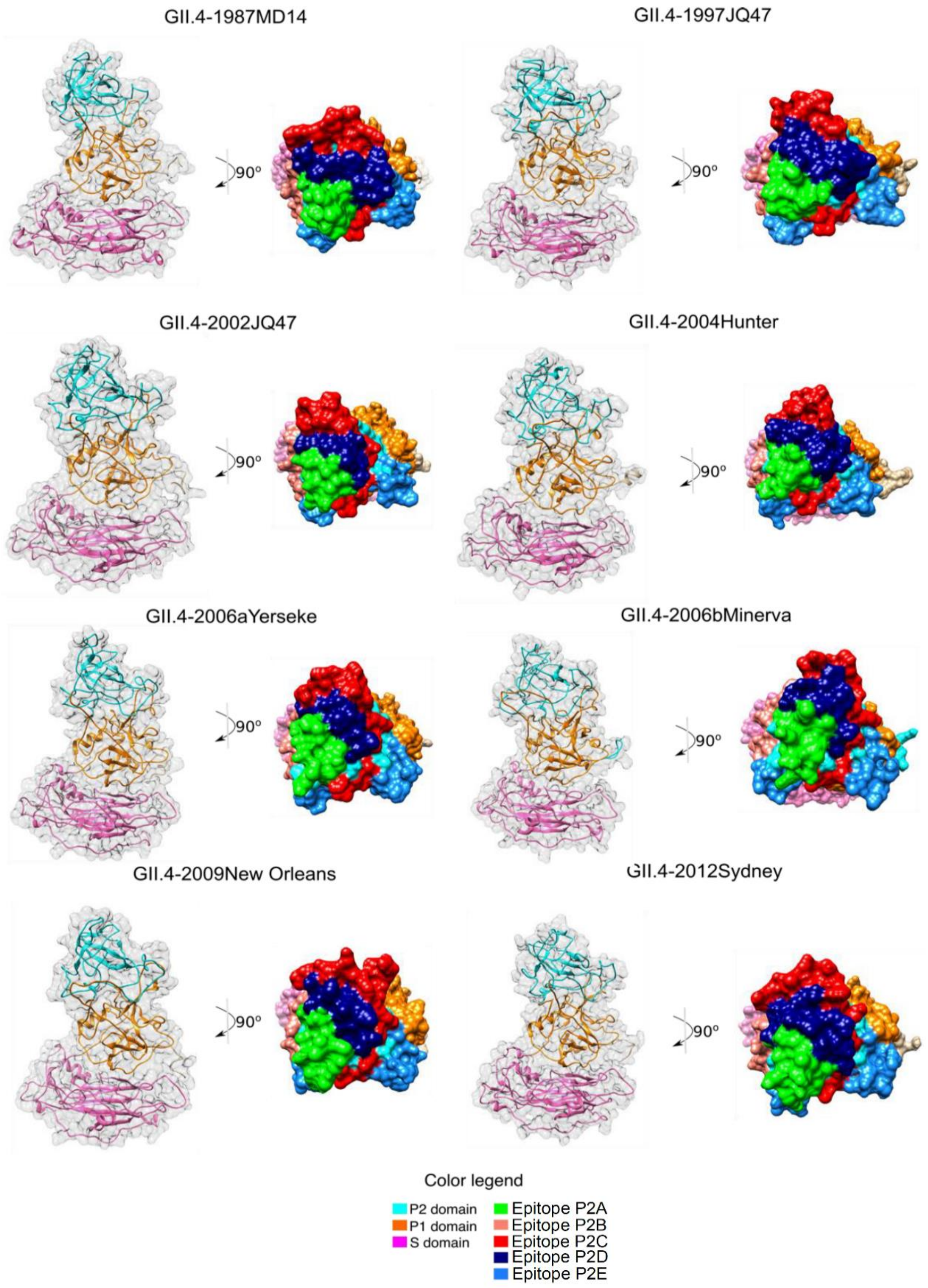

Figure 3. Three dimensional surface plots depicts the GII.4.VP1 structures color coded according to domain architecture (left side) and epitopes in P2 domain (right side).
